# Re-Examination of the Microstructural Evolution in Undercooled Co-18.5at.%B Eutectic Alloy

**DOI:** 10.3390/ma15041315

**Published:** 2022-02-10

**Authors:** Yixuan He, Yuhao Wu, Fan Bu, Yiyuan Zhang, Yifan Zhang, Bo Hei, Jianbao Zhang, Haifeng Wang

**Affiliations:** 1 State Key Laboratory of Solidification Processing, Northwestern Polytechnical University, Xi’an 710072, China; 13084838083@163.com (Y.W.); 18709251269@163.com (F.B.); yiyzhang1208@mail.nwpu.edu.cn (Y.Z.); yifanzhang4018@163.com (Y.Z.); hb13007933889@163.com (B.H.); jianbaozhang@mail.nwpu.edu.cn (J.Z.); 2 Center of Advanced Lubrication and Seal Materials, Northwestern Polytechnical University, Xi’an 710072, China; 3 Collaborative Innovation Center of NPU, Shanghai 201108, China

**Keywords:** solidification pathway, Co–B system, phase selection, pseudoeutectic regions, recalescence degree

## Abstract

The undercooling (∆*T*) dependencies of the solidification pathways, microstructural evolution, and recalescence behaviors of undercooled Co-18.5at.%B eutectic alloys were systematically explored. Up to four possible solidification pathways were identified: (1) A lamellar eutectic structure consisting of the FCC–Co and Co_3_B phase forms, with extremely low Δ*T*; (2) The FCC–Co phase primarily forms, followed by the eutectic growth of the FCC–Co and Co_2_B phases when Δ*T* < 100 K; (3) As the Δ*T* increases further, the FCC–Co phase primarily forms, followed by the metastable Co_23_B_6_ phase with the trace of an FCC–Co and Co_23_B_6_ eutectic; (4) When the Δ*T* increases to 277 K, the FCC–Co phase primarily forms, followed by an FCC–Co and Co_3_B eutectic, which is similar in composition to the microstructure formed with low Δ*T*. The mechanisms of the microstructural evolution and the phase selection are interpreted on the basis of the composition segregation, the skewed coupled zone, the strain-induced transformation, and the solute trapping. Moreover, the prenucleation of the primary FCC–Co phase was also detected from an analysis of the different recalescence behaviors. The present work not only enriches our knowledge about the phase selection behavior in the undercooled Co–B system, but also provides us with guidance for controlling the microstructures and properties practically.

## 1. Introduction

As a ubiquitous phenomenon in nature, solidification plays a critical role in metallurgy processing and it determines the final performance of the products [1]. Most solidification behaviors take place outside of the equilibrium state because of the rapid cooling rate or the large undercooling (∆*T*), which result in various microstructures and properties [2,3]. So far, the microstructural evolution and phase selection behaviors of undercooled melts have been investigated in many systems, such as the Mn–Si [4] and Cu–Sn [5] systems, using cyclic overheating combined with glass fluxing [6], directional solidification [5], and laser melting [7], with in situ observations [8] or numerical simulations [9], to manipulate and control the microstructures and properties. For instance, the uniform nature and refinement of the grains can be obtained as the ∆*T* increases, which can effectively improve the yield strength [10] and the physical properties [11]. The L2_1_-type phases in the Ni–Mn–*X* phases (*X* = In, Sn, Sb) are in favor of the enhancement of the magnetic-induced shape memory effect properties [12]. Therefore, it is of great importance to theoretically understand the microstructural evolution and phase selection behaviors during the nonequilibrium solidification.

There are mainly four types of intermetallic compounds in *M*–B systems (*M* = Fe, Co, Ni): *M*B, *M*_2_B, *M*_3_B, and *M*_23_B_6_, whose formations are dependent on the solidification routes [13,14], which are affected by the composition and technology parameters, such as the cooling rate, the undercooling, the holding time, etc., and which show different magnetic properties because of the different contents of boron. It is worth noting that the Co_23_B_6_ metastable phase is usually regarded as a good candidate for enhancing the soft properties [15], and, in addition, the Co_2_B phase with the higher boron content has an intrinsic hardness of about 816 HV, which is comparable to the hard chromium (860 HV). Its high hardness makes it a potential wear-resisting protective coating [16]. In a word, the Co–B system also shows great potential for application if the phase and morphologies can be tuned properly.

Therefore, in the next step, we focused on research about the manipulation mechanisms of the microstructure evolution and the phase selection during the solidification process in the Co–B system, with the aim of manipulating the types and morphologies of the phases to achieve the expected properties. For a better comparison, the research on the solidification behaviors of the Fe–B and Ni–B systems is briefly interpreted. As is well known, *M*–B systems (*M* = Fe, Co, Ni) are good candidates for investigating the microstructural evolution and phase selection behaviors since the ∆*T* dependencies of the morphologies, such as dendrite, lamellar eutectic, anomalous eutectic, etc., and the phases, including solid solutions and intermetallic compounds, are not only manifold, but are also involute. So far, many studies have been conducted mainly on the bases of competitive nucleation and competitive growth. The Δ*T* dependencies of the solidification pathways are found to be relatively unequivocal in the Fe–B and Ni–B systems. For example, with an increase in the Δ*T*, the solidification products change from the stable Fe_2_B phase to the metastable Fe_23_B_6_ phase, and then to the metastable Fe_3_B phase [17]. Quirinale et al. [18] studied the in situ solidification process, which is dependent on the cooling rate, in electrostatically levitated Fe_83_B_17_ alloys, and the results show that the growth of the equilibrium Fe_2_B/α-Fe phases was suppressed, which resulted in the formation of the metastable Fe_23_B_6_ phase when the cooling rate was greater than 60 K/s. For the Ni–B systems, Xu et al. [19] found that the α-Ni + Ni_3_B lamellar/anomalous eutectics formed at a low Δ*T* of 75 K in the Ni-15.6at.% B alloys, while the metastable eutectic reaction (L→α-Ni + Ni_23_B_6_) occurred when the Δ*T* increased to 262 K. A more precise critical Δ*T* was estimated to be 157 K for the competitive nucleation between the Ni_23_B_6_ and Ni_3_B phases [20,21]. However, up to now, the microstructural evolution and phase selection behaviors of the Co–B systems have still not been clarified, and many conflicting reports can be found. For example, Wei et al. [22] found that when Δ*T* > 60 K, the metastable Co_23_B_6_ phases nucleate preferentially, unlike the Co_3_B phases in the Co_79.3_B_20.7_ alloy, which can be preserved at room temperature if the cooling rate is larger than 25 K min^−1^ [23]. Li et al. [24] studied the Δ*T* dependence of the microstructural evolution of a Co_75_B_25_ alloy and found that only the α-Co and Co_2_B phases were observed over all of the achieved Δ*T* ranges. In the recent work of Liu et al. [14], up to five solidification pathways were revealed in the solidification of an undercooled Co_79.3_B_20.7_ alloy, and the primary phase changes from Co_3_B to Co_2_B to Co_23_B_6_, and then to a α-Co + Co_3_B eutectic as the Δ*T* increases. However, the mentioned pathways above were not observed in the alloy with the composition of Co_80_B_20_ [13], which is similar to the composition of Co_79.3_B_20.7_. Instead of Co_2_B, Co_23_B_6_, and α-Co + Co_3_B eutectics, the primary α-Co phase was found when the Δ*T* > 120 K. Obviously, the microstructural evolution and phase selection behaviors are still a controversial issue and are over-dependent on the alloy composition.

In the current work, the Co-18.5at.%B eutectic alloys were solidified by glass fluxing methods to systematically study the ∆*T*-dependent microstructural evolution and phase selection behaviors. We not only focused on the competition of the primary phases, but we also studied the phase selection behaviors of the residual liquid. The ∆*T*-dependent solidification pathways for the Co-18.5at.%B eutectic alloy are identified, and the formation mechanism of the FCC-Co + Co_2_B (FCC-Co + Co_3_B) eutectic, with small or ultrahigh ∆*T*, are discussed from several aspects, including the composition segregation, the skewed coupled zone, the strain-induced transformation, and the solute trapping. Moreover, the prenucleation of the primary FCC–Co phase was also detected from the analysis of the different recalescence behaviors.

## 2. Materials and Methods

The Co-18.5at.%B eutectic master alloys were prepared by arc-melting a mixture of cobalt tablets and boron blocks, with purities higher than 99.99%. The alloys were melted under an argon atmosphere in a water-cooled copper crucible, where the titanium getters were melted first to absorb the rest oxygen. Each master ingot of 50 g was turned over and remelted at least four times to ensure the chemical homogeneity. The final mass loss of each ingot was found to be within ±0.15 g. The obtained ingots were then cut into small pieces, with an average mass of about 3 g, which were to be used as the sample candidates for the undercooling experiments. One of them was analyzed by a scanning electron microscope (SEM) and electron backscatter diffraction (EBSD) to observe the as-cast microstructures.

The quartz tubes and the sample candidates for the undercooling experiments were placed in an alcoholic solution and were washed by ultrasonic waves to avoid potential heterogeneous nucleation substrates. The B_2_O_3_ flux was placed above the sample in the quartz tube to remove the impurities and to prevent the sample from oxidizing. The B_2_O_3_ flux was dehydrated at 1050 K, for 6 h in advance, to remove the gas and moisture. The quartz crucible was inserted within the high-frequency induction coil of a vacuum chamber, which was located in an in situ observation facility, for rapid solidification. After two rounds of evacuation, to a pressure of 3.0 × 10^−3^ Pa, the vacuum chamber was back-filled by a high-purity argon gas to a pressure of 5 × 10^−2^ MPa. The temperature of the melt during solidification was monitored by a two-color pyrometer, and the accuracy for the measured temperature is within ±1 K. The samples were first heated to 800 K and were held therein for 10 min to completely melt the B_2_O_3_ flux. After that, the samples were overheated to about 1800 K and were held therein for 10 min (the eutectic point, *T*_E_, is about 1406 K). Then the power was switched off to allow the undercooled melt to cool down naturally. Each sample was cyclically heated and cooled down several times until the desired undercooling was obtained. The solidification process was recorded in situ by an infrared temperature measurement system: Raytek MR1SB (Santa Cruz, CA, USA).

The as-solidified samples were prepared following the standard metallographic procedures, i.e., hot mounting in resin, grinding with 220#-to-4000# sandpapers, and polishing. The phase morphologies and constitutions, as well as the relevant orientation relationships, were characterized by SEM and EBSD, which were attached to the FEI Quanta 650F (Hillsborough, OR, USA). The transmission electron microscopy (TEM) lamellas were cut from the prepolished surface by using a dual-beam-focused ion beam (FIB) workstation (FEI Helios NanoLab 600). The TEM observations were conducted under a JEOL JEM-2200FS (JEOL, Tokyo, Japan) microscope.

## 3. Results

### 3.1. Original Microstructure and Phase Constitution

**Figure 1:**
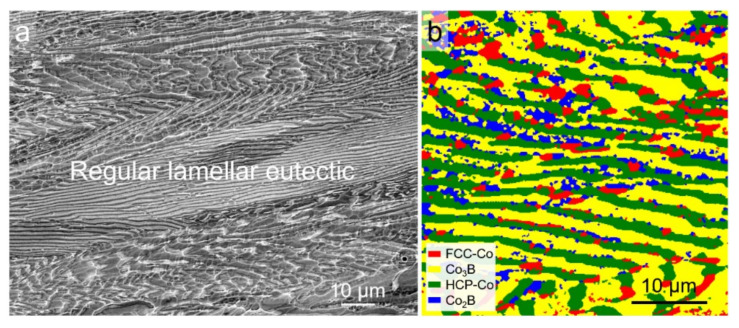
Initial microstructure of the as-cast Co-18.5at.%B eutectic alloy: (**a**) SEM-BSE image; (**b**) EBSD phase map.

The microstructure and phase constitution of the as-cast Co-18.5at.%B eutectic alloy are presented in Figure 1. It can be seen that the master alloy used here has a complete lamellar structure, with a lamellar spacing of about 3 μm, and it consists of the FCC/HCP–Co and orthogonal Co_3_B phases. In cases of near-equilibrium solidification, the coupling growth of the two phases determines the fully lamellar eutectic microstructures. The HCP–Co phase is the result of the solid-state phase transformation from the FCC–Co phase after solidification [25]. Moreover, a small amount of the tetragonal Co_2_B phase can also be detected in Figure 1b, which is likely due to the eutectoid decomposition of the Co_3_B phase at about 1118 K [26].

### 3.2. Cooling Histories

**Figure 2:**
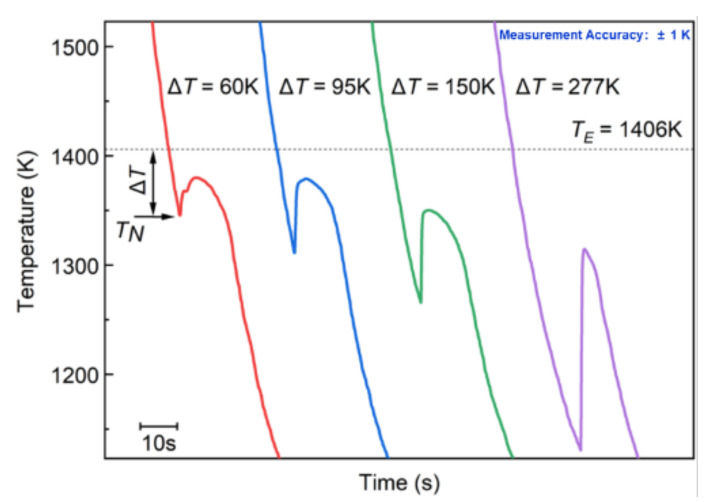
Cooling histories of rapid solidification of Co-18.5at.%B eutectic alloys with different undercoolings. Dashed line shows the eutectic temperature, *T*_E_, which is taken from a thermodynamic assessment of the Co–B phase diagram [26].

Figure 2 shows the natural cooling curves of the undercooled Co-18.5at.%B eutectic alloy, with an ∆*T* = 60 K, an ∆*T* = 95 K, an ∆*T* = 150 K, and an ∆*T* = 277 K. The undercooling (∆*T*) is defined as the difference between the congruent eutectic temperature, T_E_, and the initial nucleation temperature, T_N_, which is indicated by the black arrow. For Δ*T* = 60 K, two recalescence events are available: the overheated melt is cooled with a first recalescence event at an T_N_ = 1346 K, and it then experiences the second recalescence event. Generally, the two recalescence events should correspond to two different crystallization behaviors, which will be analyzed hereinafter, in combination with the morphologies. The recalescence degree, Δ*T*_R_, is about 32 K, and is defined as the temperature increase from the T_N_ to the maximum arrest temperature, T_R_, which can be used to predict the solid fraction for the solidification of undercooled melts [27]. After that, the temperature decreases monotonously, which indicates the end of the solidification process, and the beginning of the solid-state phase transformations, e.g., Co_3_B→FCC-Co+Co_2_B and FCC-Co→HCP-Co may occur during cooling [28,29].

For Δ*T* = 95 K, Δ*T* = 150 K, and Δ*T* = 277 K, only one recalescence event was observed during the cooling processes. Two possibilities may result in the disappearance of the second recalescence event: (1) Only one transformation process takes place during solidification; (2) The two transformation processes may share the same feature in the temperature increase, which results in the overlap of the recalescence peaks [13]. These two possibilities will be proven through the as-solidified microstructures later. The thermal-plateau time was found to be shortened, which could be attributed to the larger nucleation rate and the more homogeneously distributed nuclei, as well as to the larger growth driving force, which is due to the lower growth temperature [30].

### 3.3. Microstructures

**Figure 3:**
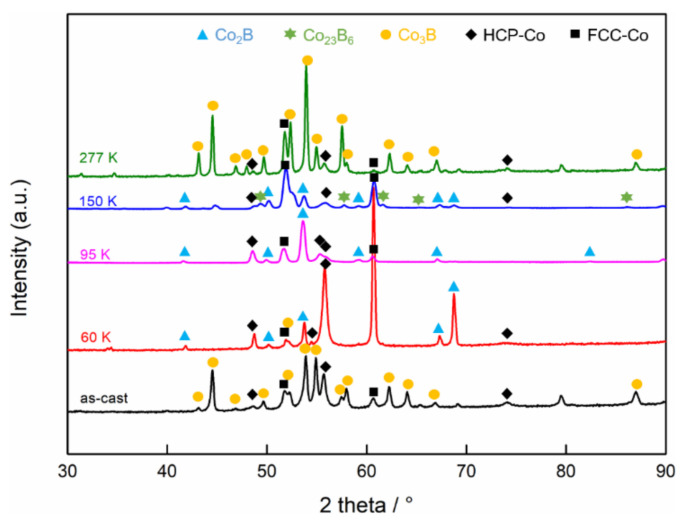
The typical XRD spectrums of the Co-18.5at.%B eutectic alloys solidified at different undercoolings.

**Figure 4:**
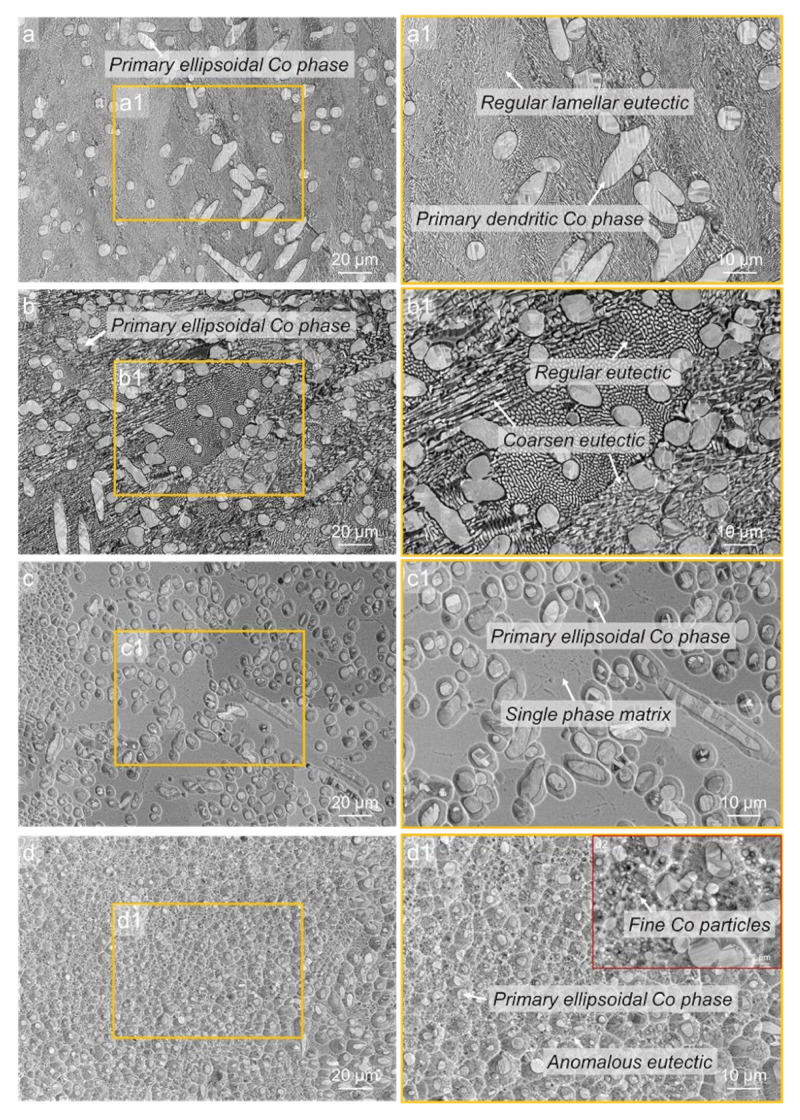
The typical microstructures of the Co-18.5at.%B eutectic alloys solidified at different undercoolings: (**a**) Δ*T* = 60 K; (**b**) Δ*T* = 95 K; (**c**) Δ*T* = 150 K, and (**d**) Δ*T* = 277 K; (**a1**–**d1**) are the enlarged regions indicated by the saffron solid squares in Figure 4a–d, respectively. The insert in the top right corner of Figure 4d1 is the magnified graph of the anomalous eutectic containing submicron-sized FCC–Co particles.

The typical XRD spectrums of the Co-18.5at.%B eutectic alloys with different undercoolings are shown in Figure 3. The as-cast sample consists of the FCC/HCP–Co and orthorhombic Co_3_B phases, and, as the undercooling increases, the Co_3_B phase is substituted by the tetragonal Co_2_B phase at the small undercoolings of 60 K and 95 K, and then an FCC phase (Co_23_B_6_ phase proved by further TEM analysis later) takes place when the Δ*T* = 150 K, and Co_3_B forms again at an ultrahigh undercooling of 277 K. To better understand the microstructure evolution as the undercooling increases, Figure 4 depicts the morphology evolution of the Co-18.5at.%B alloys with different undercoolings. For Δ*T* = 60 K, the primary dendritic or ellipsoidal structures of the FCC–Co phases are surrounded by fine regular lamellar eutectics, and the lamellar eutectics are predominant, as is seen in Figure 4a,a1. The ellipsoidal FCC–Co is a result of the dendrite fragmentation caused by the remelting because of the recalescence. In the case of Δ*T* = 95 K, the primary FCC–Co dendrites are fully broken, and parts of the growing coupled eutectic lamella are slightly coarsened, as is indicated in Figure 4b,b1. As the Δ*T* increases further to 150 K, the microstructure is characterized by a dispersion of the primary FCC–Co phases in an unknown matrix (Figure 4c). Closer observation (Figure 4c1) reveals that the matrix should only consist of a single phase, and that it exhibits features similar to the typical intermetallic compounds [23]. A similar microstructure was also found in our previous work [31], and the unknown matrix turned out to be the metastable Co_23_B_6_ phase. At the maximal undercooling obtained in the present work (Δ*T* = 277 K), the microstructures evolve into the finer globular FCC–Co phases, with smaller sizes and larger fractions, surrounded by the anomalous eutectic containing submicron-sized FCC–Co particles. In such cases, all of the FCC–Co dendrites are remelted because of the enhanced heat release, as is seen in Figure 4d. It should be pointed out that, for the representative microstructures with four different undercoolings shown in Figure 4, the primary FCC–Co phase and the second phase of either the eutectics or the single matrix coexist, which should correspond to two different recalescence events. However, for Δ*T* = 95 K, Δ*T* = 150 K, and Δ*T* = 277 K, only one recalescence event can be observed in Figure 2, which indicates that the two transformation processes share the same feature: a temperature increase, which results in the overlap of the recalescence peaks.

**Figure 5:**
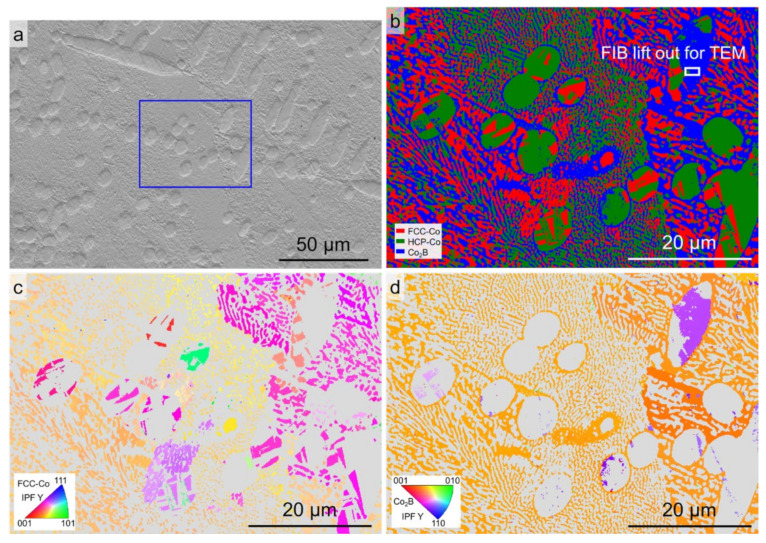
EBSD analysis of the Co-18.5at.%B eutectic alloys solidified with Δ*T* = 60 K: (**a**) SEM-ETD image showing the region for analysis; (**b**) EBSD phase map; (**c**) and (**d**) the corresponding *Y* axis inverse pole figure (IPF) maps for the FCC–Co and Co_2_B phases, respectively (the *Y* axis is parallel to the direction of gravity).

The microstructures were further analyzed by EBSD to confirm the phase constitutions and the relevant orientation relationships. Figure 5 shows the EBSD phase map and the orientation maps for the Co-18.5at.%B eutectic alloys solidified with Δ*T* = 60 K. The SEM-ETD image shown in Figure 5a indicates that the primary FCC–Co phase and the refined lamellar eutectic colonies were selected, as denoted by the blue box. Figure 5b represents the phase constituents, which exhibited the FCC–Co, HCP–Co, and Co_2_B phases in the analyzed area marked by the red, green, and blue colors, respectively. The phase constitution of the lamellar eutectic colonies is unexpectedly indexed as FCC–Co/HCP–Co + Co_2_B, rather than as FCC–Co/HCP–Co + Co_3_B, with an average confidence index above 95%. The majority of the FCC–Co phase in the primary phase is transformed to HCP–Co. In addition, one should note that a number of Co_2_B grains can be found in the ellipsoidal primary phase, which is also observed in [31]. In such cases, no Co_3_B grains are detected. Figure 5c,d shows the EBSD inverse pole figure (IPF) maps for the FCC–Co and Co_2_B phases. Several eutectic colonies can be discerned, and the constituent phases of the lamellar eutectics grow cooperatively since the eutectic orientation is fixed. The development of ellipsoidal FCC–Co phases could be attributed to the dendrite fragmentation during the post-recalescence stage since the neighboring grains share the same orientation.

**Figure 6:**
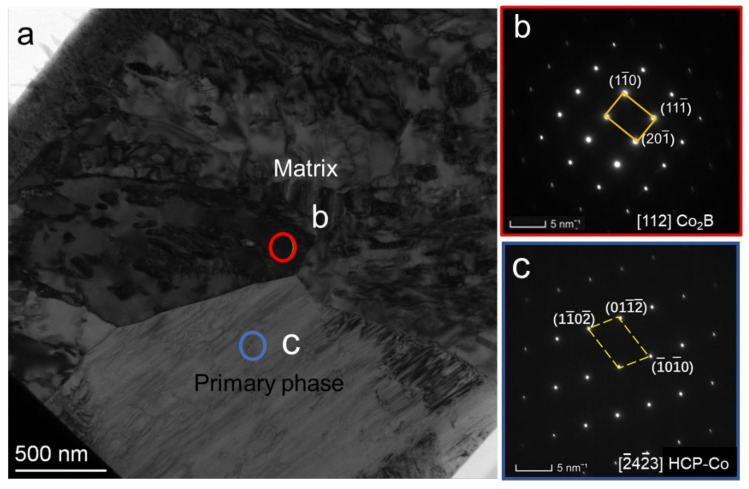
TEM analysis of a FIB lift-out lamella prepared from the location in Figure 5b: (**a**) TEM bright-field image; (**b**,**c**) SAD patterns taken from the regions indicated by circles in Figure 6a. The indices in the bottom right corner indicate the corresponding zone axes.

The Co_2_B matrix is characterized in greater detail by using TEM analysis in order to ensure the phase type. Figure 6 displays the TEM analysis of the FIB lift-out lamellar prepared from the location shown in Figure 5b. Figure 6a presents a TEM bright-field image, showing a lower magnification overview. The primary phase corresponds to the bottom half of the image, and the matrix corresponds to the top half of the image. The selected area diffraction (SAD) patterns for the two regions are shown in Figure 6b,c, which demonstrate that the matrix is in the orthorhombic Co_2_B phase, with a = b = 0.51 nm and c = 0.41 nm, which is consistent with the result of JCPDS: 25-0241.

**Figure 7:**
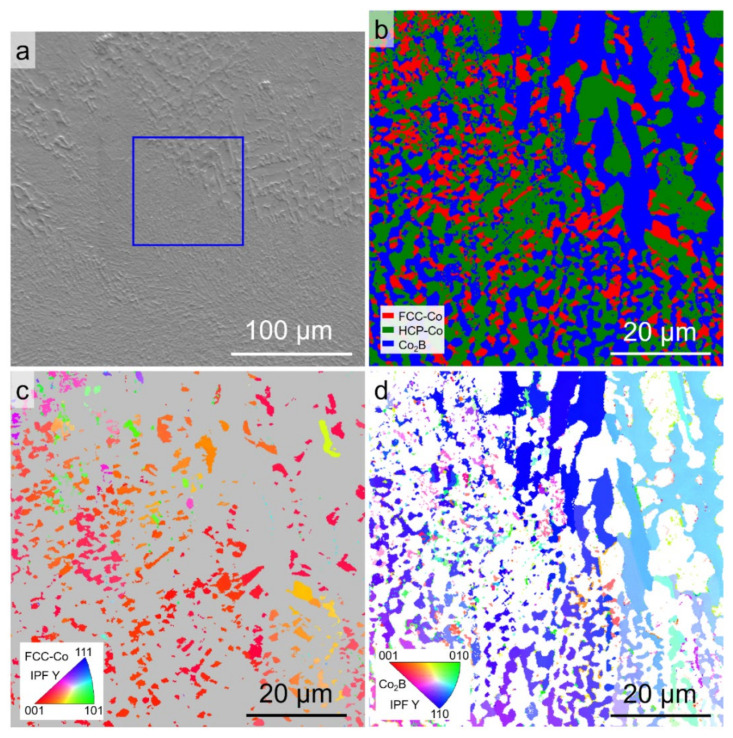
EBSD analysis of the Co-18.5at.%B eutectic alloys solidified with Δ*T* = 95 K: (**a**) SEM-ETD image showing the region for analysis; (**b**) EBSD phase map; (**c**) and (**d**) the corresponding *Y*-axis IPF maps for the FCC–Co and Co_2_B phases, respectively (*Y* axis is parallel to the direction of gravity).

Figure 7 shows the EBSD phase map and the orientation maps for the Co-18.5at.%B eutectic alloys solidified with Δ*T* = 95 K. Figure 7a indicates that both the coarsened eutectic and the fragmented anomalous eutectic were selected, as is denoted by the blue box. The phase constituents of the eutectics are still indexed as FCC–Co/HCP–Co + Co_2_B. Figure 7c,d shows the EBSD IPF maps for the FCC–Co and Co_2_B phases. The orientations of the FCC–Co and Co_2_B phases in the fragmented anomalous eutectic region are slightly deflected because of the remelting caused by the large recalescence, while the orientations of the FCC–Co and Co_2_B phases in the coarsened eutectic appear to have not been affected.

**Figure 8:**
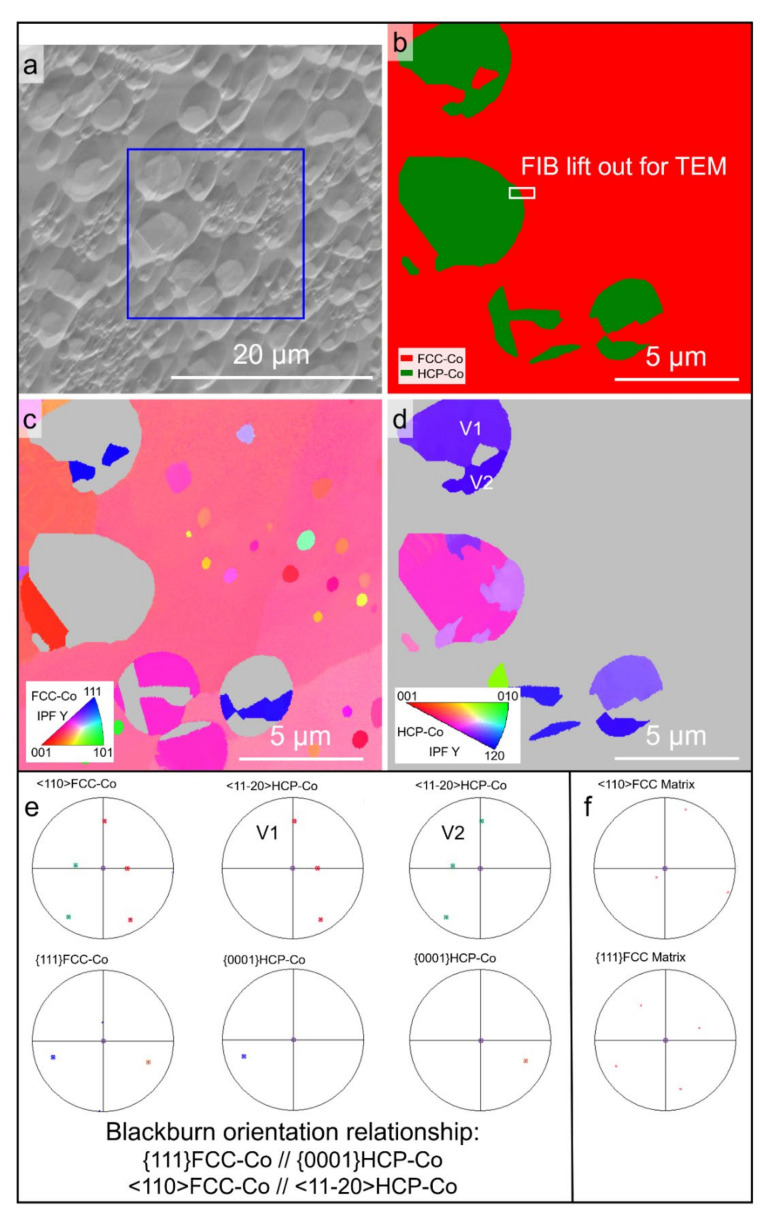
EBSD analysis of the Co-18.5at.%B eutectic alloys solidified with Δ*T* = 150 K: (**a**) SEM-ETD image showing the region for analysis; and (**b**) EBSD phase map. The small white rectangle in Figure 6b indicates the location for preparing an in-depth TEM lamella by FIB; (**c**) and (**d**) the corresponding *Y*-axis IPF maps for the FCC–Co and HCP–Co phases, respectively (*Y* axis is parallel to the direction of gravity); (**e**) orientations of FCC–Co and the associated HCP–Co in the granular primary phase plotted onto the pole figures, revealing the orientation relationship between them; (**f**) the corresponding orientation of the FCC single-phase matrix. The purple cross represents the direction of gravity.

Figure 8 shows the EBSD phase map and the orientation maps for the Co-18.5at.%B eutectic alloys solidified with Δ*T* = 150 K. Figure 8a indicates that the single-phase matrix and the primary FCC–Co phase are both involved. The single-phase matrix is unsurprisingly indexed as FCC–Co, as is shown in Figure 8b, which was also encountered in our previous work [31]. The single-phase matrix should be the Co_23_B_6_ phase. The Co_23_B_6_ and FCC–Co phases have the same space group of 225, and the lattice parameter of the Co_23_B_6_ unit cell happens to be three times that for the FCC–Co, indicating that the two phases periodically repeat the same geometric symmetrical structure, which results in misindexing during the EBSD characterization. The misindexing of the Co_23_B_6_ phase is further confirmed hereinafter using TEM. Figure 8c,d shows the EBSD IPF maps for the FCC–Co/ Co_23_B_6_ matrix and the HCP–Co phases. The neighboring ellipsoidal primary FCC–Co phases should originate from the fragmentation of one dendrite since they share the same orientation. The IPF map for the FCC Co_23_B_6_ matrix is colorful, which indicates that the Co_23_B_6_ matrix is texture-free. The matrix exhibits the coarsened equiaxial Co_23_B_6_ phase with a slight trace of the FCC–Co + Co_23_B_6_ eutectic because some fine FCC–Co particles can be found in the matrix (Figure 8c). The HCP–Co phase, which results from the partial solid-state phase transformation of FCC–Co, is only found within the ellipsoidal primary phase. Two variations and the corresponding pole figures are taken as an example to indicate that the Blackburn orientation relationship is obeyed between the parent FCC–Co and the associated HCP–Co (Figure 8e). Figure 8f shows the corresponding pole figures for the FCC Co_23_B_6_ matrix next to the two HCP–Co variations, and no fixed orientation relationship can be found among them.

**Figure 9:**
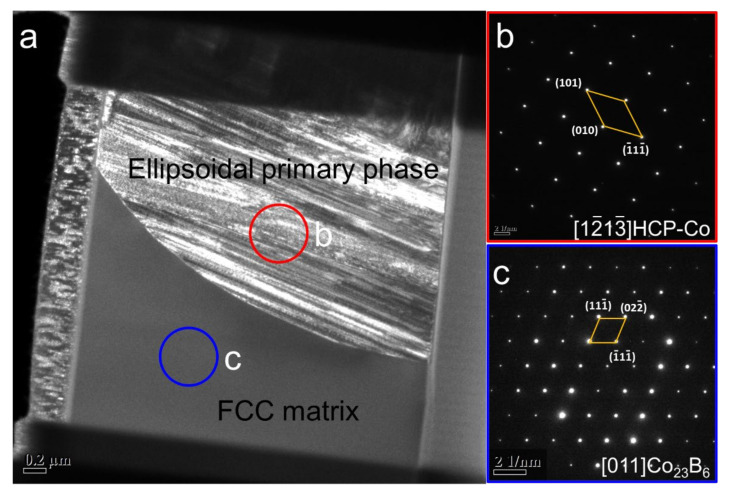
TEM analysis of a FIB lift-out lamella prepared from the location in Figure 6b: (**a**) TEM bright-field image; (**b**,**c**) SAD patterns taken from the regions indicated by circles in Figure 7a. The indices in the bottom right corner indicate the corresponding zone axes.

The FCC Co_23_B_6_ matrix is characterized in greater detail by using TEM analysis to verify and distinguish the FCC–Co and Co_23_B_6_ phases. Figure 9 displays the TEM analysis of the FIB lift-out lamella prepared from the location shown in Figure 8b. Figure 9a presents a TEM bright-field image, showing a lower magnification overview. The ellipsoidal primary phase corresponds to the top half of the image, and the FCC matrix corresponds to the bottom half of the image. The selected area diffraction (SAD) patterns for the two regions are shown in Figure 9b,c, which demonstrates that the FCC matrix is actually the Co_23_B_6_ phase.

**Figure 10:**
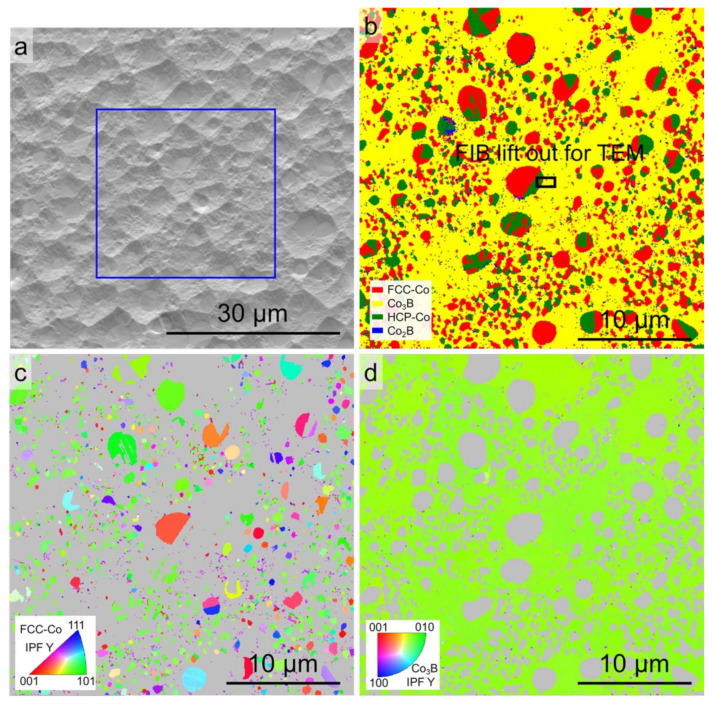
EBSD analysis of the Co-18.5at.%B eutectic alloys solidified with a Δ*T* = 277 K: (**a**) SEM-ETD image showing the region for analysis; (**b**) EBSD phase map; (**c**) and (**d**) the corresponding *Y* axis IPF maps for the FCC–Co and Co_3_B phases, respectively (*Y* axis is parallel to the direction of gravity).

Figure 10 shows the EBSD phase map and the orientation maps for the Co-18.5at.%B eutectic alloys solidified with Δ*T* = 277 K. Figure 10a indicates that both the granular primary phase and the anomalous eutectic were selected, as is denoted by the blue box. In this case, the phase constituents of the eutectics are indexed as FCC–Co/HCP–Co + Co_3_B, which is the eutectic structure obtained under the near-equilibrium condition. Figure 10c,d shows the EBSD IPF maps for the FCC–Co and Co_3_B phases. The orientations of the FCC–Co and Co_3_B phases in the anomalous eutectic region appeared to be well-orientated, which might be due to the lower *T*_R_ achieved by the recalescence (Figure 2).

**Figure 11:**
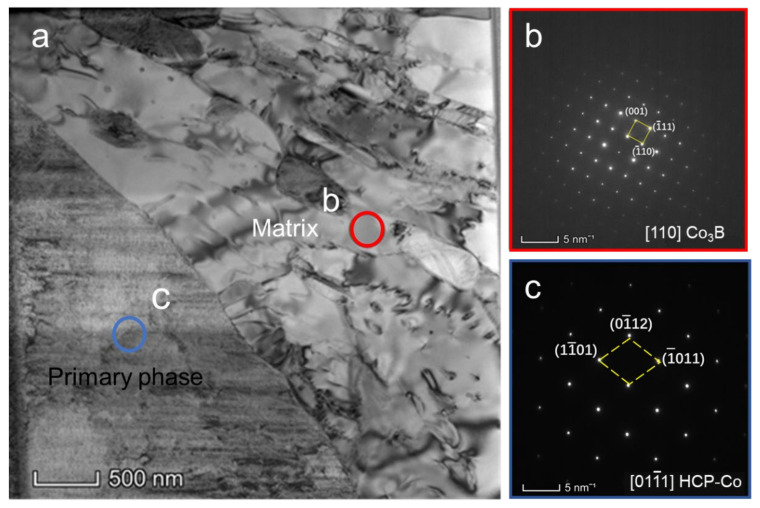
TEM analysis of a FIB lift-out lamella prepared from the location in Figure 10b: (**a**) TEM bright-field image; (**b**,**c**) SAD patterns taken from the regions indicated by circles in Figure 11a. The indices in the bottom right corner indicate the corresponding zone axes.

The Co_3_B matrix is also further ensured by the TEM analysis. Figure 11 displays the TEM analysis of the FIB lift-out lamella prepared from the location shown in Figure 10b. Figure 9a presents a TEM bright-field image. The selected area diffraction (SAD) patterns for the two regions are shown in Figure 11b,c, which demonstrates that the matrix is in the orthorhombic Co_3_B phase, with a = 0.51 nm, b = 0.66 nm, and c = 0.47 nm, which fits well with the result of JCPDS: 12-0443.

On the basis of the foregoing characterization results, we conclude that there are at least four possible solidification pathways for the Co-18.5at.%B eutectic alloy: (1) The lamellar eutectic structure (Figure 1), which consists of the FCC–Co and Co_3_B phase forms, under equilibrium or near-equilibrium solidification conditions, with extremely low Δ*T*, since the coupled growth of the eutectic is easily broken with the increasing Δ*T* [13]. As a result, the FCC–Co phase precipitates as the primary phase; (2) The FCC–Co phase primarily forms, followed by the eutectic growth of the FCC–Co and Co_2_B phases when the Δ*T* is relatively low (Δ*T* < 100 K) (Figure 4a,b, Figure 5 and Figure 6); (3) As the Δ*T* increases further, the FCC–Co phase primarily forms, followed by a metastable Co_23_B_6_ phase, with a trace of an FCC–Co and Co_23_B_6_ eutectic, rather than an FCC–Co and Co_2_B eutectic (Figure 8 and Figure 9); (4) When the Δ*T* increases to a certain degree, e.g., 277 K in the present work (Figure 10), the FCC–Co phase primarily forms, followed by the FCC–Co and Co_3_B eutectic, which is similar to the composition of the microstructure formed with low Δ*T*. The difference between them lies in the fact that the types of eutectics are different. The FCC–Co and Co_3_B eutectic, formed with low Δ*T*, is a regular lamellar eutectic, whereas the eutectic formed with Δ*T* = 277 K is an anomalous eutectic, which is characterized by a dispersion of the fine globular FCC–Co particles in the Co_3_B matrix.

## 4. Discussion

### 4.1. Formation of FCC–Co/Co_2_B Eutectic with Δ*T* Less Than 100 K

According to the phase diagram of the Co–B system [26], the Co-18.5at.%B alloy is at the equilibrium eutectic point, which should be constituted by the FCC–Co and orthogonal Co_3_B phases. However, in the present work, the eutectic is composed of the FCC–Co and tetragonal Co_2_B phases, rather than the FCC–Co/Co_3_B eutectic forms, following the primary FCC–Co phase when the Δ*T* < 100 K. Despite the fact that this phenomenon has been observed and verified in the Co–B system by many researchers [14,24], the formation mechanism is still controversial. Li et al. [24] thought that the suppression of the peritectic reaction of liquid and Co_2_B into Co_3_B could result in the formation of an FCC–Co/Co_2_B eutectic in the Co_75_B_25_ alloy. Liu et al. [14] attribute the formation of the FCC–Co/Co_2_B eutectic in the Co_79.3_B_20.7_ alloy to the remelting and resolidification of the Co_23_B_6_ phase, i.e., the Co_23_B_6_ phase primarily forms, but it is subsequently remelted and resolidified, along with the remaining liquid, into the FCC–Co/Co_2_B eutectic when 85 K < Δ*T* < 281 K. In our previous work [31], the reason of the formation of the FCC–Co/Co_2_B eutectic is that the composition of the remaining liquid becomes extremely rich in boron because of the massive precipitation of the primary Co with the application of the magnetic field. In this work, although no magnetic field was applied, the volume fraction (*f*_s_) of the precipitated FCC–Co phase is still large, e.g., *f*_s_ (Δ*T* = 60 K) = 27%, and *f*_s_ (Δ*T* = 95 K) = 39%, indicating that, in these cases, the composition of the remaining liquid remains rich in boron. This may make a contribution to the formation of the FCC–Co/Co_2_B eutectic, although it may not be the main reason.

In the present work, since the FCC–Co/Co_2_B eutectic is formed from the residual liquid, the theories on the suppression of the peritectic reaction (L + Co_2_B → Co_3_B) [24] and the remelting and resolidification of the Co_23_B_6_ phase [14] are supposed to be inappropriate. Therefore, the concept of a eutectic coupled zone is employed herein to obtain a decent explanation. The eutectic coupled zone representing the growth temperature/composition region is where an entirely eutectic microstructure forms [32]. 

It is well known that there are two types of eutectic coupled zones: the symmetric coupled zone and the skewed coupled zone. A symmetric coupled zone generally exists in a eutectic system of the nonfaceted/nonfaceted type. A skewed coupled zone is often found in a eutectic system of the faceted/nonfaceted type, and it is normally skewed towards the faceted phase owing to its persistent growth difficulties. Moreover, the growing rate of the phases will be suppressed significantly when undercooling is ultrahigh because of the sluggish kinetic diffusion [32], i.e., the skewed coupled zone is a finite scope above a certain nucleation temperature, T_R_.

**Figure 12:**
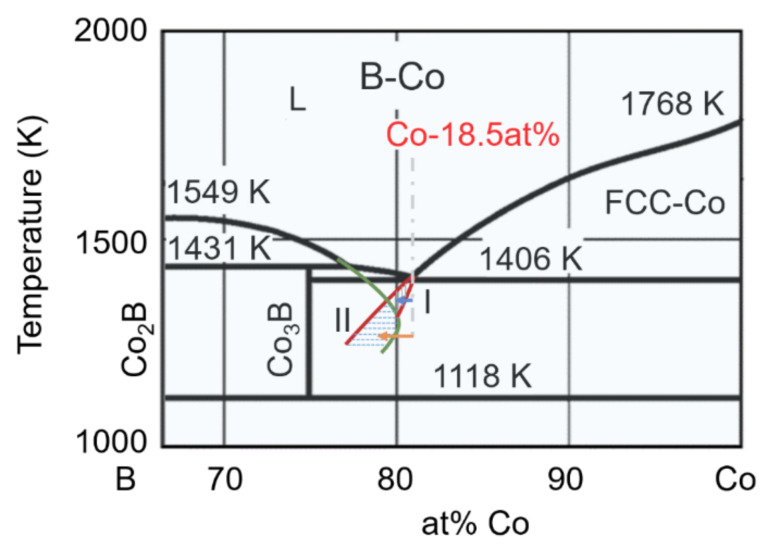
Schematic Co-rich region of the Co–B phase diagram showing the skewed eutectic coupled zones.

It has been proven that skewed coupled zones exist in the Co–B eutectic system [13]. The growth difficulties associated with the faceted Co_3_B and Co_2_B phases, as compared to the solid-solution of the FCC-Co phase, result in the eutectic coupled zones skewing towards the faceted Co_3_B and Co_2_B phases. Figure 12 shows a part of the phase diagram of a Co–B alloy with skewed coupled zones. The first coupled zone skewed toward the faceted Co_3_B phase is made up of the extension of the liquidus lines of the Co_3_B and FCC–Co phases, which is named after Region I, as is shown in Figure 12. The second coupled zone (Region II), which is skewed toward the faceted Co_2_B phase, is composed of the extension of the liquidus lines of the Co_2_B and FCC–Co phases. For an alloy with the composition of Co-18.5at.%B (grey dashed dotted line), solidification occurs via a normal phase transformation process, according to the Co–B phase diagram, when the Δ*T* is very low, i.e., when the liquid solidifies into the FCC-Co/Co_3_B eutectic phases simultaneously under the near-equilibrium condition. With the Δ*T* slightly increases, the primary FCC–Co phase forms preferentially, since the FCC–Co solid solution phase invariably has a priority in the nucleation, compared to the intermetallic compounds, such as Co_3_B, Co_2_B, and Co_23_B_6_ [13]. The precipitation of the primary FCC–Co phase ensures that the residual liquid is rich in boron, i.e., the composition of the residual liquid shifts into the first skewed coupled zone (Region I), as is indicated by the blue arrow. As a result, in such a case, the primary FCC–Co combined with the FCC–Co/Co_3_B eutectic should be obtained. Unfortunately, this very low Δ*T* is hard to achieve under the current experimental conditions. With the Δ*T* increasing further, e.g., to 60 K, the composition segregation of the residual liquid caused by the precipitation of the primary FCC–Co phase shifts into the second skewed coupled zone (Region II), as is indicated by the orange arrow, which results in a final solidified microstructure that is made up of the primary FCC–Co and FCC–Co/Co_2_B eutectic. It is worth mentioning that, despite the fact that the skewed eutectic regions are not calculated accurately herein, they do exist in the Co–B systems, and they could be a probable explanation for the formation of the FCC–Co/Co_2_B eutectic. To verify this assumption, an intensive study of the calculation of the skewed coupled zones in the Co–B system could be a focus in future work.

It is important to note that the strain-induced transformation may also contribute to the formation of the FCC–Co/Co_2_B eutectic to some extent. Wang et al. [33] studied the influence of the pressure on the phase stability and found that, under a high pressure of 80 kbar, the Co_2_B phase, rather than the Co_3_B phase, was obtained in the Co_80_B_20_ amorphous alloy because the pressure causes volume shrinkage, which favors the formation of a Co_2_B phase with a high density. During the recalescence, in addition to the remelting effect as the temperature increases, the driving pressure for the fluid flow could also increase dramatically [34]. Liu et al. [35] argued that the origin of the strain effects could be attributed to the transfer from the volume mobility to the interdendritic transport of the melt through the crisscrossing solid skeleton obtained after dendritic coherency, where the materials start to develop strength. Hunt also points out that this volume change could contribute to the negative pressure near the S/L interfaces, which could lead to the formation of microcavities [36], the collapse of which produces extremely high positive pressures during recalescence. Therefore, the accumulated stresses in the primary dendrite network during rapid solidification may stimulate the formation of the FCC–Co/Co_2_B eutectic. A further experimental verification and an in-depth analysis are required to confirm this assumption.

### 4.2. Formation of FCC–Co/Co_3_B Anomalous Eutectic with High Δ*T*

When the Δ*T* is extremely high (277 K), the final solidification microstructure is made up of the granular primary FCC–Co phase and the FCC–Co/Co_3_B anomalous eutectic, which is similar to the microstructure achieved when the Δ*T* is very low. This may be attributable to the appearance of the solute trapping caused by the extremely high Δ*T*. When the Δ*T* is extremely high, the primary FCC–Co dendrite is supersaturated with more solute. Moreover, the solidification time is significantly shortened compared to the low Δ*T*. As a result, the diffusion of the atoms is largely weakened, which causes the effect of solute trapping, with no deviation in the composition [37]. In our previous work, the FCC–Co and Co_3_B phase grows faster than that in the low Δ*T*, at approximately 1.4 m/s and 0.8 m/s [13], respectively, which increases the partition coefficient, *K*_V_, and the liquid and solid compositions could be similar. It is noted that the enlarged difference in the growth velocity between the FCC–Co and Co_3_B phases could effectively manipulate the morphologies; i.e., the coupling growth mechanism is broken thoroughly, and the regular lamellar eutectic is transferred to the anomalous eutectic. The great heat release remelts the FCC–Co phases into the dispersed particles embedded in the Co_3_B matrix. A similar phenomenon was also reported in Co–Si alloys [38] under nonequilibrium solidification.

### 4.3. Prenucleation of Primary FCC–Co Phase

**Figure 13:**
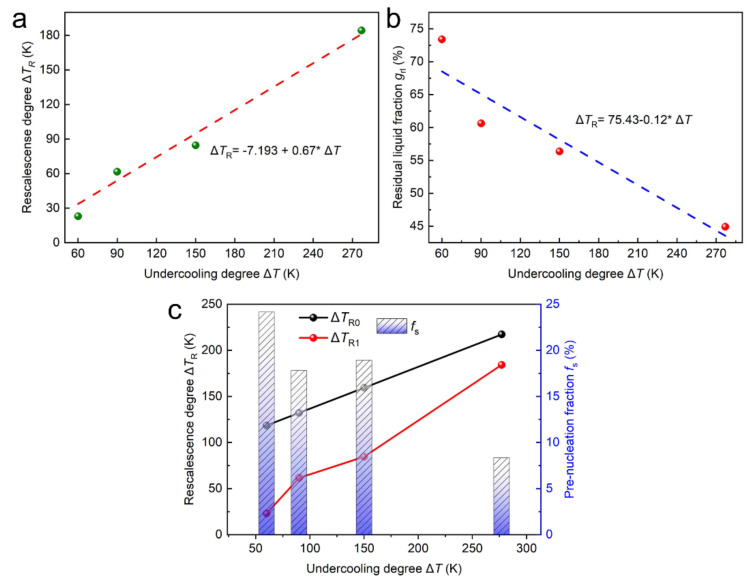
The undercooling Δ*T* dependencies of the recalescence degree, Δ*T*_R1_: (**a**) residual liquid fraction, *g*_rl_; and (**b**) the calculated prenucleation fraction, *f*_s_, in (**c**) the Co_81.5_B_18.5_ eutectic alloys.

It is acknowledged that large amounts of the primary FCC–Co phases are found in all the final solidification microstructures with different Δ*Ts*. However, the recalescence degree, Δ*T*_R_, which is obtained from the undercooling curves, is not actually so conspicuous, and the Δ*T*_R_ acquired from the undercooling curves is the superposition heat release of several phases. For convenience, we think that the Δ*T*_R_ represents the heat release of the primary FCC–Co, since the recalescence and heat release of the primary FCC–Co phase is predominant within the extremely short solidification time obtained from the cooling curves. For example, when the Δ*T* = 60 K, the volume fraction of the primary FCC–Co phase is approximately 25%, but the temperature increases because the latent heat release is only 22 K. Generally, the recalescence degree, Δ*T*_R_, is in proportion to the nucleation and the growth. The larger Δ*T*_R_ is related to the shorter thermal-plateau time, and the greater nonequilibrium solid fraction during recalescence, according to the dendric fragmentation model [39]. The Δ*T*-dependent recalescence degree, Δ*T*_R1_, is depicted in Figure 13a, where the scattered points were obtained experimentally, and the solid lines are the fitted results. It is clear that the recalescence degree, Δ*TF*_R1_, increases with the increase in the Δ*T* because of the enhanced nonequilibrium effect. The calculated critical hypercooling Δ*T* is about 395 K when all the melt solidifies during the recalescence, and there is no residual liquid that can be solidified into a secondary phase (*g*_rl_ = 0). The obtained Δ*T* is not beyond 277 K, which indicates that our experimental condition is nonadiabatic. Hence, the heat release compared to the temperature increase during recalescence cannot be neglected. The fraction of the residual liquid *g*_rl_-dependent Δ*T*_R_, with consideration to the prenucleation, can be expressed by Equation (1) [40]:

(1) $$g_{\mathrm{rl}}\approx 1-\frac{C_{P}}{\Delta H_{m}}\left(\Delta T_{R}+\phi t\right)-f_{s}\left(T\right)$$

where the *C*_p_ and Δ*H*_m_ are the thermal capacity and the latent heat of the fusion, respectively (*C*_p_ = 30.6 J/mol/K [41], and Δ*H*_m_ = 16.06 KJ/mol [22] for the primary FCC–Co phase); *t*, which is equal to approximately 0.3 s, is the recalescence time, which is much larger than those for the pure metals or the solid solution alloys (0.001–0.01 s); ϕ is the cooling rate, which is about 20 K/s in the present work; and $f_{s}\left(T\right)$ is a function of the temperature, which denotes the fraction of prenucleation.

Figure 13b shows the Δ*T* dependence of the fraction of the residual liquid, which was obtained from the statistical analysis of the SEM morphologies shown in Figure 3. With the enhancement of the Δ*T*, the recalescence degree increases, and the residual liquid after recalescence is reduced. Therefore, the theoretical recalescence degree, Δ*T*_R0_, could be obtained according to Equation (1), combined with the statistical result shown in Figure 10b, and the assumption that *f*_s_(*T*) = 0. The Δ*T*_R0_ shown in Figure 10c is larger than the Δ*T*_R1_, and the difference decreases as the Δ*T* increases. This may be, in part, because the experimental condition in the present work is nonadiabatic. Another possible reason is the pre-nucleation [42] of the primary phase. It is well known that the atomic bonds of crystals are only partly broken upon melting, and there are a lot of short-range orderings (SROs) that correspond to the solid crystal in the melts within a wide temperature range above the *T*_m_ [43]. The SROs that change with the overheating temperature are metastable, and the liquid–liquid structure transition (LLST) [44,45,46] can be induced when the overheating temperature exceeds a critical value [47]. These SROs could be acting as the sites for nucleation, which may increase the volume fraction of the primary FCC–Co, but they do not contribute to the temperature increase during recalescence. The *f*_s_(*T*) in this work can be calculated by Equation (2):

(2) $$f_{s}\left(T\right)=\frac{C_{P}}{\Delta H_{m}}\left(\Delta T_{R0}-\Delta T_{R1}\right)$$

The calculated *f*_s_(*T*) is shown in Figure 13c with the blue-column diagram. The *f*_s_(*T*) decreases as the Δ*T* increases, which indicates that the intrinsic existing SROs causing the prenucleation decrease with the increasing Δ*T*. In the present work, in order to achieve a high Δ*T*, normally a higher overheating temperature is required. In such cases, the application of higher overheating temperatures may induce the LLST, resulting in the consumption and dissolution of the SROs. On the contrary, when a low Δ*T* is expected, a smaller overheating temperature is usually exerted. As a result, the liquid is full of SROs since it has not experienced the LLST. This may be why the Δ*T*_R1_ obtained from the cooling curves is lower than the theoretical Δ*T*_R0_.

It is worth noting that we have put forward the idea that the retained FCC–Co clusters within the heterogeneous melts could affect the recalescence behaviors; yet, the degree of recalescence is actually the heat release of all the phases, and not a single phase. Here, we came up the hypothesis and performed the appropriate treatments for the convenience of analysis. However, it is more accurate that the alloy systems with simpler solidification paths and single-phase recalescence are more appropriate for that goal. In addition, the discussions still need further attention and should be verified by dedicated experiments in future work, e.g., by the systematic variation of the overheating temperature and the holding time, etc. 

## 5. Conclusions

The undercooling (∆*T*) dependencies of the solidification pathways, the microstructural evolution, and the recalescence behaviors in undercooled Co-18.5at.%B eutectic alloys were systematically explored, and the conclusions are as follows:

(1) The solidification paths are as follows: (1) The regular lamellar eutectic colonies consisting of the FCC–Co and Co_3_B phase nucleate at a near-equilibrium solidification with extremely low Δ*T*; (2) As the Δ*T* increased slightly, the Co_3_B phase was substituted by the Co_2_B phase, with a mixture of the lamellar and anomalous eutectic for Δ*T* < 100 K. (3) With the Δ*T* increasing further, the Co_2_B phase changed to the metastable Co_23_B_6_ phase for Δ*T* < 200 K, with the ripened primary α-Co ellipsoid due to the remelting effect; (4) When the Δ*T* is extremely high, the Co_3_B phase takes place again, with refined α-Co particles for Δ*T* > 200 K, where the composition is similar to that of the microstructure formed with low Δ*T*;
(2) The mechanism of the phase selection is interpreted on the basis of the composition segregation, the skewed coupled zone, the strain-induced transformation, and the solute trapping. When Δ*T* < 100 K, the Co_2_B phase nucleates preferentially within the residual liquid, which is attributed to the existence of the FCC–Co/Co_2_B pseudo-eutectic region and the accumulated strain during recalescence. Moreover, the Co_3_B phase forms at an ultrahigh Δ*T*, which results from the solute trapping during the rapid solidification;
(3) The recalescence behaviors of the primary FCC–Co phase were analyzed, and the prenucleation solid fraction, owing to the existence of solid-like clusters or SROs within the heterogenous melt, decreases as the undercooling increases and, hence, the recalescence degree rises dramatically.

## Data Availability

Data is contained within the article.
